# Rapid geographical indication of peppercorn seeds using corona discharge mass spectrometry

**DOI:** 10.1038/s41598-021-95462-0

**Published:** 2021-08-09

**Authors:** Preeyarad Charoensumran, Monrawat Rauytanapanit, Nontawat Sricharoen, Barry L. Smith, Kanet Wongravee, Simon Maher, Thanit Praneenararat

**Affiliations:** 1 Department of Chemistry, Faculty of Science, Chulalongkorn University, Phayathai Rd., Pathumwan, Bangkok, 10330 Thailand; 2 The Chemical Approaches for Food Applications Research Group, Faculty of Science, Chulalongkorn University, Phayathai Rd., Pathumwan, Bangkok, 10330 Thailand; 3 Center of Excellence in Bioactive Resources for Innovative Clinical Applications, Chulalongkorn University, Bangkok, 10330 Thailand; 4 Sensor Research Unit, Department of Chemistry, Faculty of Science, Chulalongkorn University, Phayathai Rd., Pathumwan, Bangkok, 10330 Thailand; 5 Department of Electrical Engineering & Electronics, University of Liverpool, Brownlow Hill, Liverpool, L69 3GJ UK

**Keywords:** Mass spectrometry, Sustainability

## Abstract

With increasing demands for more rapid and practical analyses, various techniques of ambient ionization mass spectrometry have gained significant interest due to the speed of analysis and abundance of information provided. Herein, an ambient ionization technique that utilizes corona discharge was applied, for the first time, to analyze and categorize whole seeds of black and white peppers from different origins. This setup requires no solvent application nor gas flow, thus resulting in a very simple and rapid analysis that can be applied directly to the sample without any prior workup or preparation. Combined with robust data pre-processing and subsequent chemometric analyses, this analytical method was capable of indicating the geographical origin of each pepper source with up to 98% accuracies in all sub-studies. The simplicity and speed of this approach open up the exciting opportunity for onsite analysis without the need for a highly trained operator. Furthermore, this methodology can be applied to a variety of spices and herbs, whose geographical indication or similar intellectual properties are economically important, hence it is capable of creating tremendous impact in the food and agricultural industries.

## Introduction

Mass spectrometry in combination with hyphenated techniques (e.g., chromatography) is the gold standard for chemical identification where legal enforcement is required^1^. Recently ambient ionization (AI) MS has been used to provide rapid assessment of authenticity in a variety of scenarios such as the adulteration of Whisky^2^, pesticide residue analysis on strawberry surfaces^3^, and the quantification of bisphenol A and its analogs contained within food packaging^4^, to mention only a few. AI approaches seek to reduce or entirely negate sample preparation, allowing analysis to be performed on samples in their native environment^5^. Popular AI sources include the likes of paper spray^6–10^, direct analysis in real time^11–13^ and desorption electrospray^14,15^. AI sources that utilize corona discharge (CD) have been extensively reported, such as ASAP^16,17^ and DAPCI^18,19^. These approaches typically rely on a heated nebulizing/desolvating gas flow in one form or another to vaporize a liquid/solid sample prior to ionization and analysis. CD without any accompanying gas flow is routinely used with ion mobility instruments^20,21^ for trace vapor detection and has been incorporated with various AI sources to improve ionization efficiency. For instance, Song et al*.* applied CD in combination with an inexpensive ultrasonic nebulizer to detect antibiotic drugs in milk^22^, Mullen et al. combined secondary electrospray with CD to improve detection of explosive compounds^23^, and Sekimoto et al*.* increased DART ionization efficiency by addition of a CD pin between the source and inlet^24^. A number of studies have utilized direct ambient CD ionization for MS studies^25–28^.

A key aim of this study was to develop a methodology for direct peppercorn seed analysis that is sufficiently robust and does not require any sample preparation nor requires the addition of any consumables, such as solvents or gas. We surmised that CD ionization can be applied to solid phase samples that naturally contain volatile compounds for direct analysis without any prior vaporization. This significantly reduces the complexity of the setup to be simply the application of a CD needle in proximity to the sample. As is shown in this study, with appropriate sample types, the information obtained from this analytical setup can have great impact to their respective fields. In this regard, aromatic spices are a perfect sample for this exploration because of the possible impact to the food and agricultural industry. For example, black pepper (*Piper nigrum*), colloquially called “King of Spices”, has evolved to be one of the most widely used spices in the world. Originating in India, black pepper has become a mainstay in both eastern and western cuisines, with its global export value reaching 1.6 billion USD in 2019^29^. Thus, various aspects of quality control of this product are vital to the sustainability of its market^30–33^. Prominent examples are the development of analytical methods^34–36^ to detect adulteration by cheaper materials, e.g., pepper husk or papaya seed, and to uncover geographical origins of peppers^37^. The latter study is directly linked to geographical indication (GI), which has become an increasingly important leveraging tool in trade negotiations^38,39^ and sales valuation of products. GI protection is worth, on average, double the value compared to non-certified products^40^. In the EU alone, the value of GI products is estimated to be worth over €75 billion^40^. Therefore, the incentive for mis-characterization of product origin is very appealing for illegitimate producers. The drive to enforce GI necessitates the development of chemical analysis methods that are rapid, non-biased and versatile^41–43^.

Herein, we demonstrate, for the first time, the application of a CD for the direct ionization of peppercorn seeds to obtain a set of mass spectrometry (MS) data that are indicative of their geographical origins. Combined with robust pre-processing and chemometric analyses (principal component analysis (PCA) and linear discriminant analysis (LDA)), this analytical method was able to distinguish the geographical origin of different sources of black peppers, along with some simpler studies including the type of peppers (black vs white), and the origin of white peppers. An iterative reformulation feature selection algorithm was used to ascertain the key *m/z*’s necessary to construct accurate classifier models. Our findings indicate that this rapid, versatile and non-targeted MS protocol has the potential to be used for further GI studies relating to other herbs, spices and foodstuffs in general.

## Material and methods

### Sample preparation

Black pepper seeds originating from Thailand (6 sources), China (1 source), India (1 source), and the United Kingdom (3 sources) were used in this study; all samples were purchased locally from supermarkets or ordered online from reputable suppliers. White peppers from Thailand (6 sources) and China (1 source) were also purchased in a similar manner. All samples were removed from their respective packaging for analysis. Otherwise, no sample pretreatment or preparation was carried out. All experiments were performed in accordance with relevant guidelines and regulations.

### Acquisition of MS data from peppers

**Figure 1:**
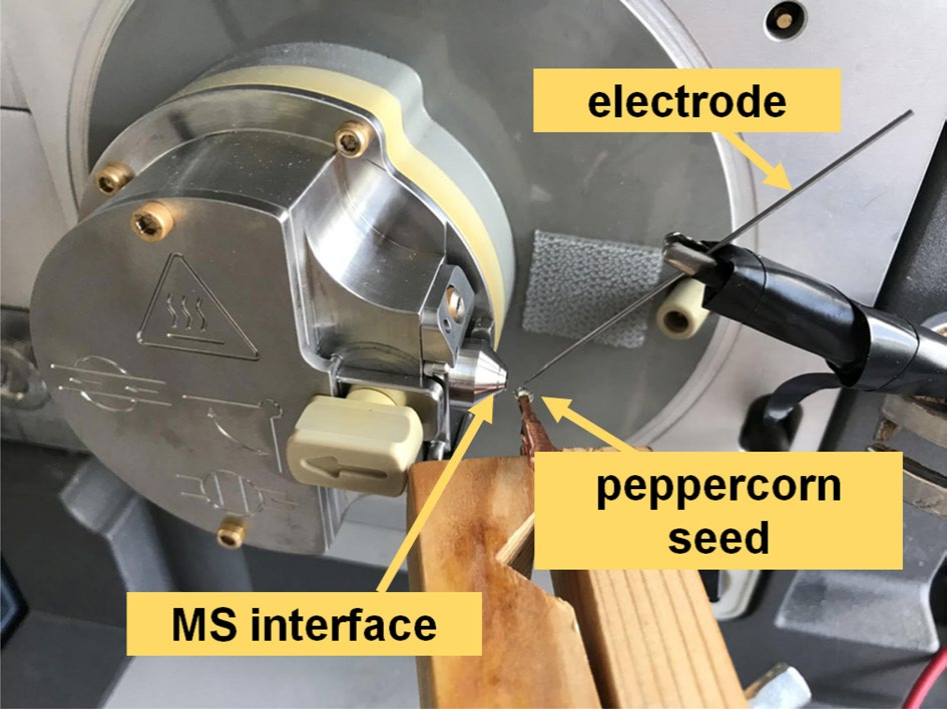
Experimental setup for direct ionization MS on peppercorn seeds.

A Waters Xevo TQ MS (Waters Corporation, Milford, MA, USA) was used in this study with the following instrument parameters: cone voltage at 20 V, source temperature at 100 °C, and acquisition mass range of 50–500 *m/z*. The experimental method is depicted in Fig. 1. Briefly, a peppercorn seed was affixed with a distance of approximately 3 mm from the MS inlet. A stainless steel needle was placed 2–3 mm above the sample with an applied potential of 3 kV, as determined by prior optimization experiments to yield a stable signal. A corona discharge (CD) develops at the apex of the needle due to the large potential and small radius of curvature which subsequently ionizes vapors from the pepper sample. Data was acquired for a duration of 2 min with 1 s per scan. At least 9 seeds from each source were tested, with a blank (no seed) being run in between different sample sources. The complete set of data can be found in the supplementary information.

### Data pre-processing for subsequent chemometric analyses

A series of automated data pre-processing steps were performed in MATLAB (Mathworks). (1) Firstly, any *m/z* values over 300 were discarded due to the lack of any significant ion peaks. (2) Mass spectra were smoothed with the weighted linear regression function within the peak width (10 data points) in order to prevent peak fluctuations in the same *m/z* channel. Baseline correction, with zeroing of any point with negative values, was performed. (3) The intensity of each *m/z* data point was scaled logarithmically in order to give each data point equal significance. (4) The data was aligned using 4 reference peaks (*m/z* = 109.1, 137.15, 177.15 and 235.3), which are consistently present in all samples and background scans, to ensure any instrument drift during analysis is corrected. (5) The MS was down sampled to 0.1 amu grid reducing the *m/z* datapoints from 3172 to 2000. (6) Local maxima within 0.8-amu windows were confirmed reducing the variables from 2000 to 267. (7) Peak intensity data from real samples were subtracted by the intensity data of the blank, i.e., the runs without any peppercorn seed but with the same setup. (8) Finally, white and black pepper datasets are combined into a single data table consisting of 267 (*m/z*’s) by 156 (sample intensities) datapoints. Further filtering of *m/z*’s closer than 0.5 amu reduces the dataset to a final 199 × 156 datapoints. An overview scheme of the data preparation with an example mass spectrum after preprocessing is illustrated in Fig. S1.

### Visualizing method and classification method

The data sets showed a very high number of variables (267 variables). To visualize and determine the number of significant factors from such high numbers, further data reduction was performed. The data was centered over all samples prior to the data reduction process. In this study, Principal Component Analysis (PCA) was used to reduce the dimensionality in a multivariate data set to those data characteristics that contribute the most to the overall variance, with the first component representing the greatest variance of the data (principal component 1—PC1), the second greatest variance being projected to the second coordinate (PC2), and so on. PCA scores can be used to visualize the clusters of samples, which share common influences. More detailed information on PCA is discussed elsewhere^44,45^. In this study, PCA was employed to visualize the underlying relationships of the data. The clusters of samples were either visualized using PCs with the maximum variances (PC1-PC3), or using the best discriminating PCs.

To obtain the classification performance, Linear Discriminant Analysis (LDA) was used for class prediction. This was done by creating a model boundary (classifier) between classes using linear discriminant function in order to define the directions in which the classes are best separated. However, in the LDA algorithm, it is necessary to calculate the inverse of the variance–covariance matrix. Therefore, if the number of variables is larger than the number of samples, the variance–covariance matrix will be a singular matrix that cannot be inverted. Hence, in this study, the discriminant features of the PCA-LDA were used as inputs to the classification model. The analysis was done by merging the modeling runs of two algorithms based on PCA to reduce the dimensionality of data matrix into 3PCs and sequentially LDA to create the classifier for class predictions of samples. The predictive ability of the developed PCA-LDA model is evaluated by “leave-one-out” cross validation (LOOCV). The mean centering is performed on the corresponding training set samples as appropriate, while the test sets are centered according to parameters obtained from the training set. The combination of validation procedures of PCA-LDA and the LOOCV approach were performed by the following steps:

1. For the data matrix (***X***), a mass spectrum of a sample (***x***_test_) was removed to be used as a test set, while the remaining mass spectra are formed as a training set (***X***_train_).
2. PCA was performed on the training set to obtain score (***T***_train_) and loading (***P***_train_). In this study, only the first 3 PCs were used to represent the overall variance of the data.
3. The score (***t***_*t*est_) of a test sample (***x***_test_) was calculated by using the pseudo-inverse of loading matrix from the training set (***P***_train_) as ***t***_test_ = ***x***_test_ ***P***^T^_train_ (***P***_train_*** P***^T^_train_)^-1^, where ^T^ is a transpose operator and ^-1^ is an inverse operator
4. The LDA classifier boundary of each class was built from ***T***_train_ to predict the class of a test sample using ***t***_test_.

The procedure is repeated until all samples have been assigned as a test sample. The contingency table can be then constructed to express the performance and stability of the developed classifier.

### Methods for identifying sets of significant variables

#### Fisher weight

Fisher weight^46^ is defined by $f_{i} = {{\left[ {\sum\nolimits_{g = 1}^{G} {I_{g} \left( {\overline{x}_{jg} - \overline{x}_{j} } \right)^{2} } } \right]} \mathord{\left/ {\vphantom {{\left[ {\sum\nolimits_{g = 1}^{G} {I_{g} \left( {\overline{x}_{jg} - \overline{x}_{j} } \right)^{2} } } \right]} {\left[ {S_{pool, j}^{2} \sum\nolimits_{g = 1}^{G} {\left( {I_{g} - 1} \right)} } \right]}}} \right. \kern-\nulldelimiterspace} {\left[ {S_{pool, j}^{2} \sum\nolimits_{g = 1}^{G} {\left( {I_{g} - 1} \right)} } \right]}}$ when there are *G* classes, where $\overline{x}_{j}$ is the mean of variable (*m/z*) *j* of all classes, *I*_*g*_ is the number of samples in class *g*, $S^{2}_{{{\text{pool}}\;j}}$ the pooled standard deviation. The calculation is based on the ratio of within-class variance to between-class variance. It shows a main advantage over *t*-statistic as it can be further use for the data with > 2 classes. However, when it is simplified for a two-class model (*G* = 2), the rank of the variables is the same as the *t*-statistic although there is no sign. The *m/z* values with the highest values of fisher weight (rank number 1) are considered as potential candidates to be used as geographical markers.

#### Iterative reformulation of training sets

This technique^47^ was performed by randomly selecting 70% of the entire dataset as a training set for several times (100 in this case). Each training may thus result in different significant *m/z* values being selected. If the same *m/z* value is chosen many times as a geographical marker for each split in the data, it indicates that such a data point is likely a robust and authentic marker. In each iteration, the optimized number of ranked data points on Fisher weight scores are recorded as the most significant variables. The procedure was repeated 100 times, thus obtaining 100 lists of significant *m/z* data points. Any *m/z* values which presented in all iterations were identified as impactful geographical markers.

## Results and discussion

### Features of MS data obtained from direct ionization of black peppers

**Figure 2:**
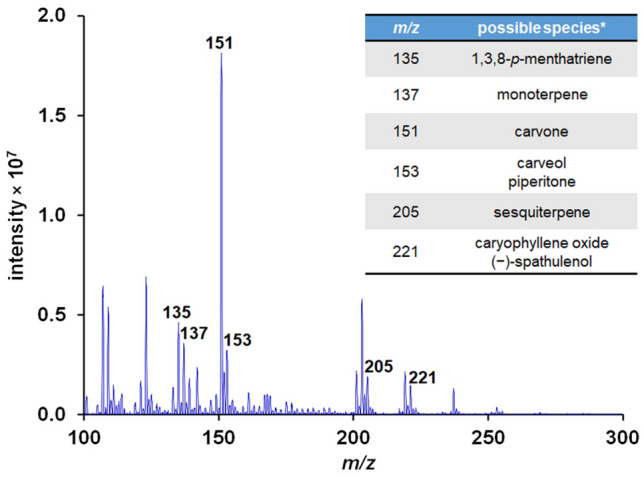
A representative MS spectrum from the sample BP(Thailand) – 06, along with some examples of putative chemical species that were deduced from the literature^50–53^.

An example spectrum (Fig. 2) highlighted some interesting features from the MS data obtained. First, it is surprising, at first glance, that a peak corresponding to piperine (*m/z* 286 for [M + H]^+^) or its derivatives cannot be seen at all. Piperine is responsible for the pungency property of black pepper, and makes up to 7% of its dried weight^48^. This disappearance is likely due to the higher melting point and boiling point, as well as the higher polarity, compared to volatile terpenes, which is another group of compounds commonly found in black pepper. Moreover, in a study examining black pepper and white pepper utilizing a similar ionization mechanism, proton transfer reaction (PTR)-MS, piperine was not detected either^49^. In any case, an aqueous extract of black pepper was shown to provide a clear signal of piperine via PS-MS (Fig. S2). Since the main goal of this work is to discover a key set of data that can differentiate the origins of black peppers, the absence of a single, albeit major, compound is deemed to be inconsequential. Next, volatile terpenes were indeed clearly found in this experimental setup. For example, a peak corresponding to monoterpene (C_10_H_16_, *m/z* 137 for [M + H]^+^) can be seen. Apart from piperine, these are a group of terpenes that directly contribute to the flavor profiles of black peppers^50–53^. Examples include myrcene, sabinene, and terpinene. Also, another peak at *m/z* 205 is visible, which is attributed to sesquiterpene (C_15_H_24_). To improve the confidence of some of the suggested peak assignments, we performed some exemplary MS/MS experiments focusing on the collision-induced dissociation of *m/z* 151 and *m/z* 205. In Fig. S3, it can be seen that there is no significant difference between the CD-MS/MS spectrum from black peppercorn seed and the spectrum from a direct-infusion MS/MS experiment of (*R*)-carvone, for the parent ion *m/z* 151. Hence suggesting that the majority of the signal at *m/z* 151 is likely to be (*R*)-carvone. Likewise, agreement was found from a comparison between a CD-MS/MS spectrum of a black pepper seed and a library spectrum^54^ for *m/z* 205 (Fig. S4), which we attribute to δ-elemene. Furthermore, these suggested assignments have previously been identified as major components of pepper seed extract^50^.

Interestingly, the amounts of these compounds in each source of black pepper, as reflected in the ion intensities, can vary. For instance, while the representative sample in Fig. 2 had *m/z* 151, likely carvone, as the most intense peak, some other samples had other peaks as the major component (Figs. S5 and S6 for the complete set of MS spectra, which were plotted from the complete set of numerical data in the supplementary information). These differences, when treated with systematic statistical analysis, may be sufficient for the differentiation of black peppers from different origins.

### Data analysis of obtained MS data

**Figure 3:**
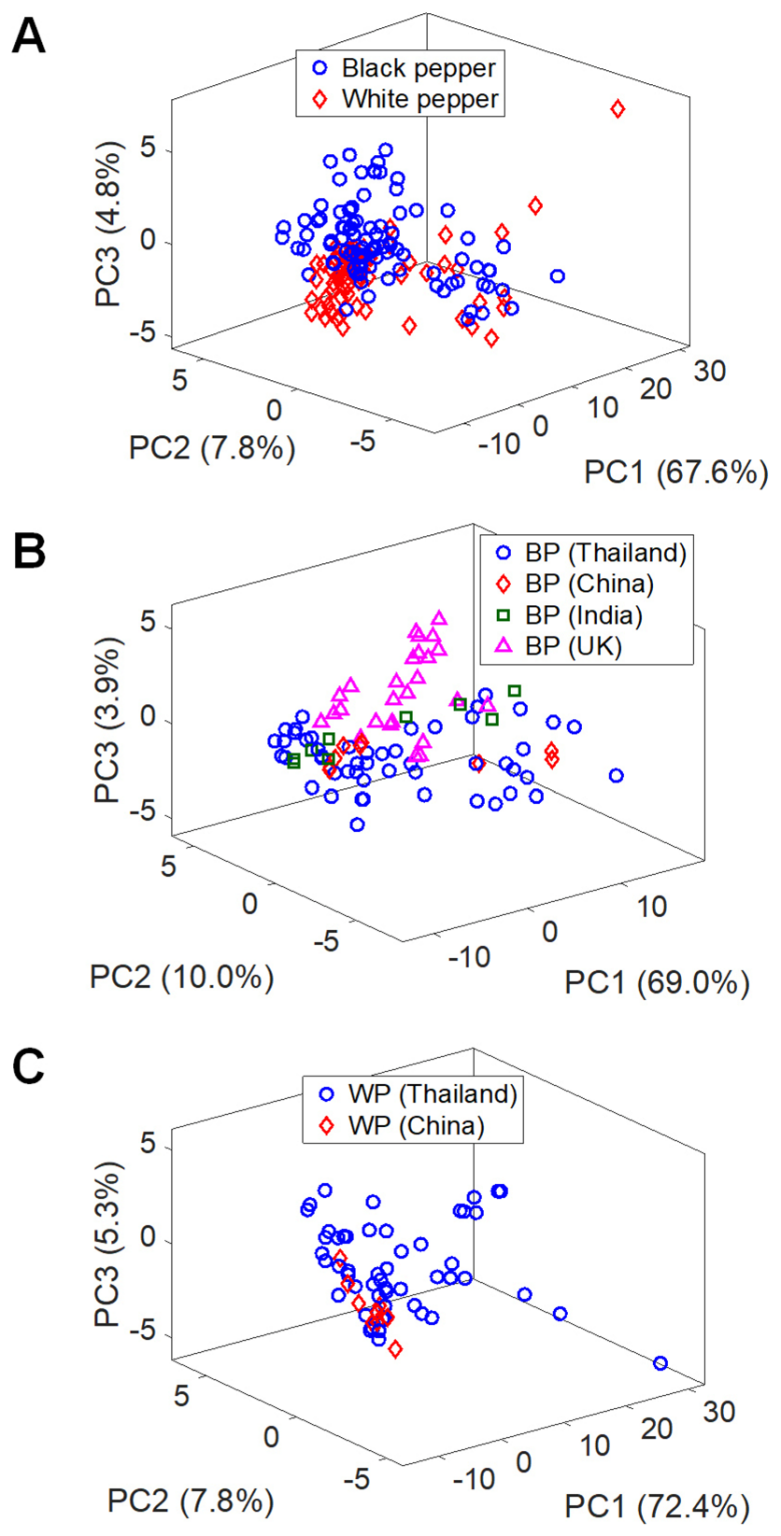
PC score plots of the first 3 principal components of the pre-processed data using all *m/z* data points to visualize the cluster relationship of (**A**) types of peppers, (**B**) origins of black peppers and (**C**) origins of white peppers.

In this study, multivariate data analysis was performed on the mass spectra in order to discriminate peppers based on three categorizations: I) the types of peppers (black and white peppers including all origins), II) the origins of black peppers (Thailand, China, India and UK), and III) the origins of white peppers (Thailand and China). First, one-sample *t*-tests were used to analyze the raw data for their variabilities in the analysis. It was found that all *m/z* data points from seeds of the same source do not show any statistical difference at the 5% significance level, i.e., accepting the null hypothesis. Thus, this indicates that variability of analysis was generally low. Then, all *m/z* data points were used for PCA analyses on the types of peppers, the origins of black peppers, and the origins of white peppers using the first three PCs (Fig. 3) with the total variance > 70% in all cases. Overall, the PCA score projections in this manner exhibit poor separation between classes in most studies. In addition, the contingency table of the classification using PCA-LDA is shown in Table S1. Classification rates of 76.92%, 74.46% and 69.35% were observed in the discrimination of the types of pepper, the origins of black peppers and the origins of white peppers, respectively. This result indicates that PCA based on the largest principal components may not always be the most effective method. In some cases, especially in biological systems^47,55^, the PCs with high variance may instead correlate with the background noise of biological samples and the best discriminator might present at the latter PC with small variance. Hence, to explore the possibility of obtaining useful information in small-intensity data points, some analysis on other PCs was conducted.

**Figure 4:**
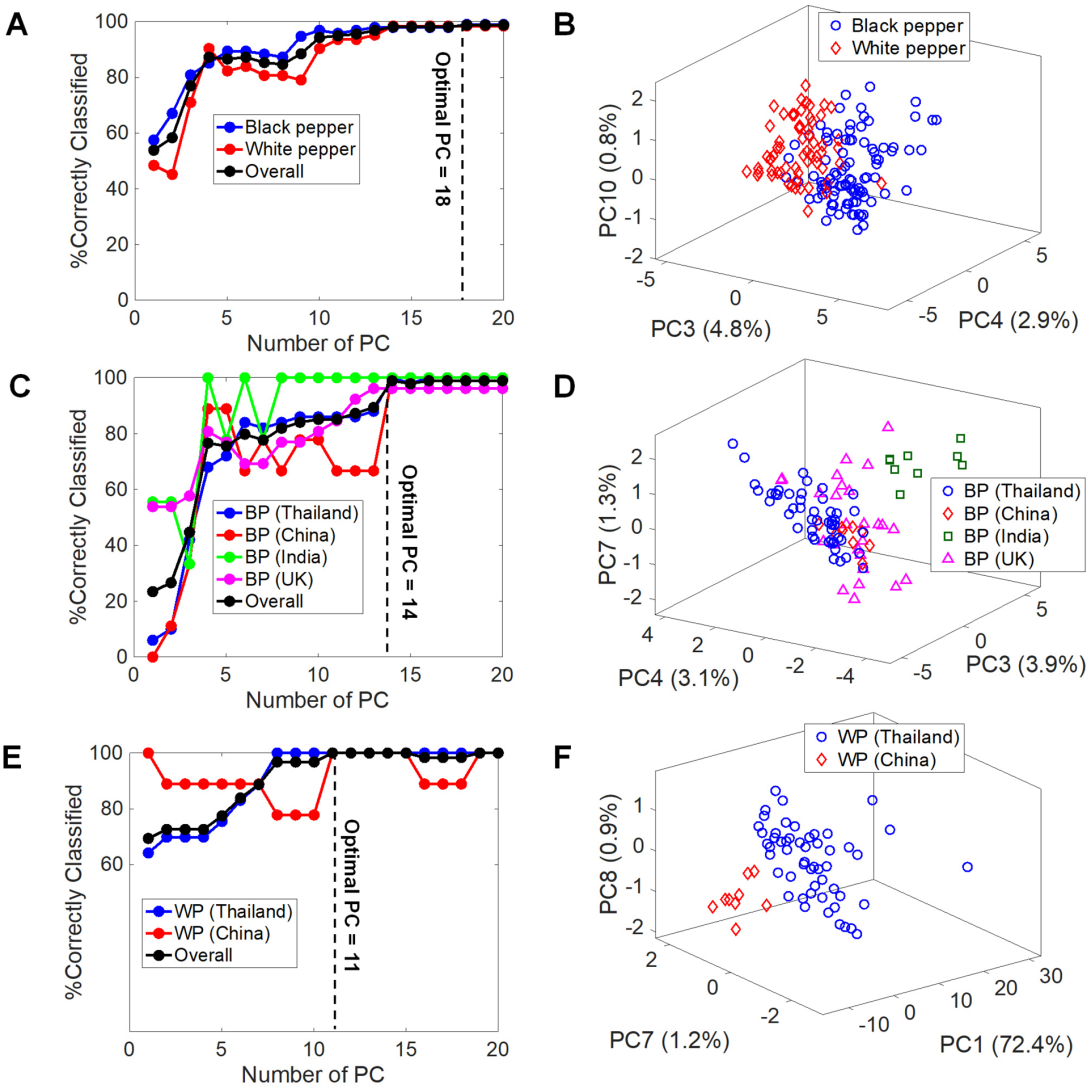
The percentage of correctly classified samples with the number of PCs used to build a model (PC1-20), along with their respective PC score plots using the best discriminate PCs for the study of (**A**) the types of peppers, (**B**) is the corresponding PC score plot, (**C**) the origins of black peppers, (**D**) is the corresponding PC score plot, and (**E**) the origins of white peppers with (**F**) showing the corresponding PC score plot.

As shown in Fig. 4 (A,C,E), it can be seen that the classification accuracy using the first three PCs is poor for all three studies. With the inclusion of latter PCs, it was revealed that 18, 14, and 11 PCs were optimal for the classification of the types of peppers, the origins of black peppers, and the origins of white peppers, respectively. In addition, the prediction strength of each PC was calculated to visualize the discrimination of the PCs in each study (shown in Fig. S7). The higher the prediction strength, the better the discrimination power. It can be seen that some latter PCs showed higher prediction strength. That is, PC 3-4-10, PC 3-4-7, and PC 1-7-8, are the sets of PCs with the highest prediction strength for the studies of the types of peppers, the origins of black peppers, and the origins of white peppers, respectively. The score plots with the PCs with the highest prediction strength are shown in Fig. 4 (B,D,F).

**Figure 5:**
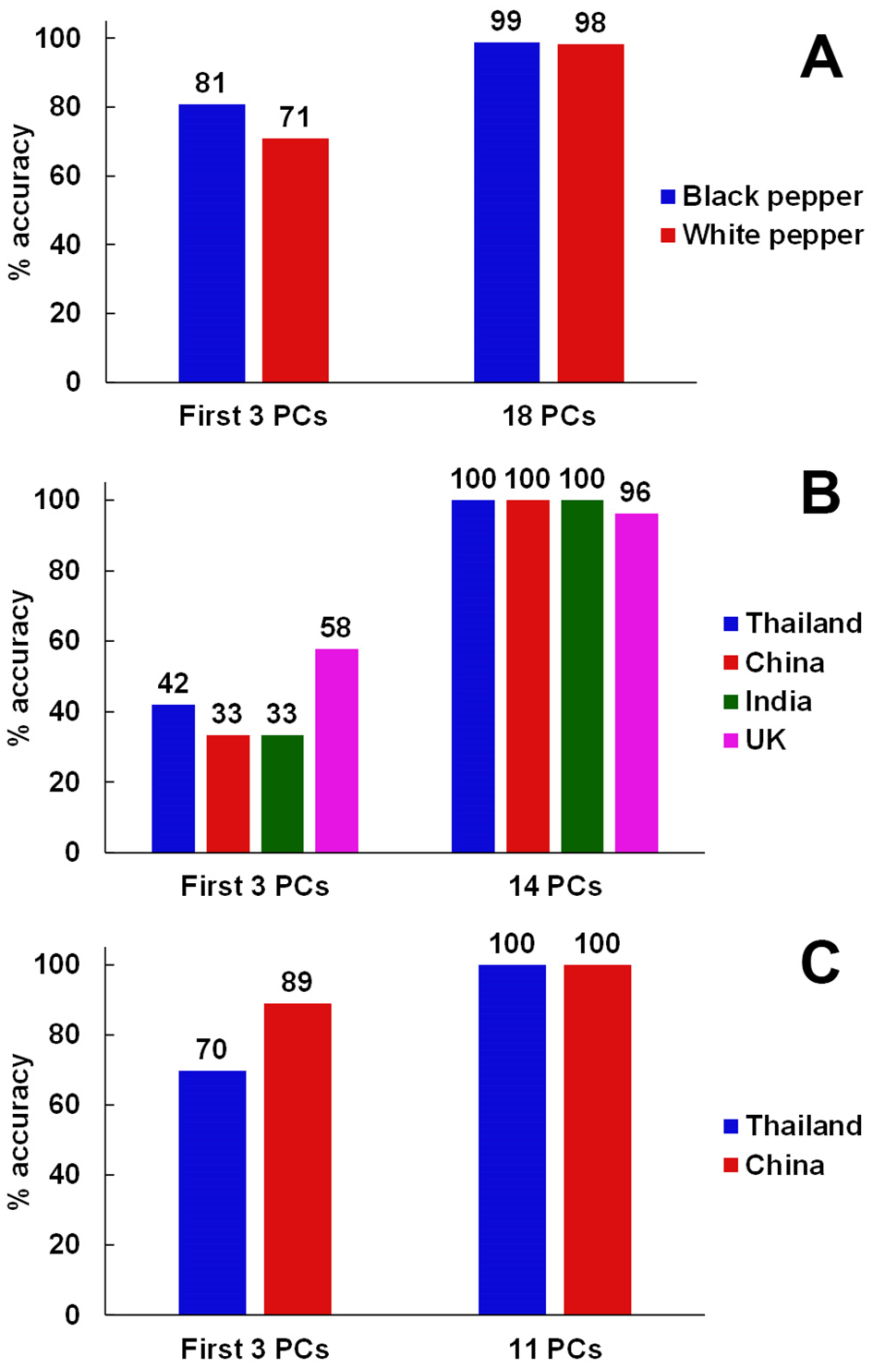
Classification accuracies of (**A**) the discrimination between black and white peppers, (**B**) the geographical discrimination of black peppers, and (**C**) the geographical discrimination of white peppers.

To provide some numerical data for comparison, the classification rates for each PCA-LDA model built from both the first three PCs and the optimal number of PCs are illustrated in Fig. 5 (Table S1 for contingency tables). The overall classification accuracies using the first three PCs are merely 76.92%, 44.68% and 72.58% for the studies of the type of peppers, the origins of black peppers, and the origins of white peppers, respectively. On the other hand, the classification rates for the cases that were built from the optimal number of PCs appear to be significantly higher, with the scores of 98.72%, 98.94% and 100%. Although the most optimal numbers of PCs for the LDA classifications were > 10 PCs in all cases, the classification rates increased dramatically only in the range of PC1-5. These results suggested that the first five PCs are the most relevant, but the latter PCs are still meaningful in differentiating between the classes. This is in good agreement with the PC score plots in Fig. 4 using the latter PCs with high prediction strength. In addition, we also evaluated the differentiation power of PS-MS data from a sample set of black peppers using their aqueous extracts. The result (Fig. S8) showed a similar trend in that multiple PCs were required to achieve 100% prediction accuracy, and that the best PCs were not from only the first three PCs. This similarity suggested that the nature of these samples, i.e., peppers, is amenable to discrimination by various MS-derived data. Thus, CD-MS, which requires no solvent and no extra sample preparation step, is an attractive and convenient method for rapid analysis to uncover geographical indication of peppers.

### Significant *m/z* data points as markers

A conventional approach for variable selection is to perform a selection method (Fisher weight in this case) on the entire dataset, and determine the significance of an *m/z* value as a marker from its magnitude of test value. However, selecting a set of markers from an entire dataset may encounter an overfitting issue. An alternative approach is to identify potential *m/z* data points from several spitted training sets. This is called as “iterative reformulation of training set models”, which was first introduced elsewhere^47^. In this study, we also employed this approach to confirm the relevance of the obtained *m/z* data points. That is, a randomized partial set (70% of the entire dataset) was used for the discovery of relevant *m/z* data points, whose appearances were then counted. After 100 iterations, all *m/z* values were then evaluated for the number of times they appeared in the list of relevant *m/z* data points of each split training set. As different *m/z* values may be differentially selected in each iteration, those data points that are most frequently selected are likely the most significant markers.

**Figure 6:**
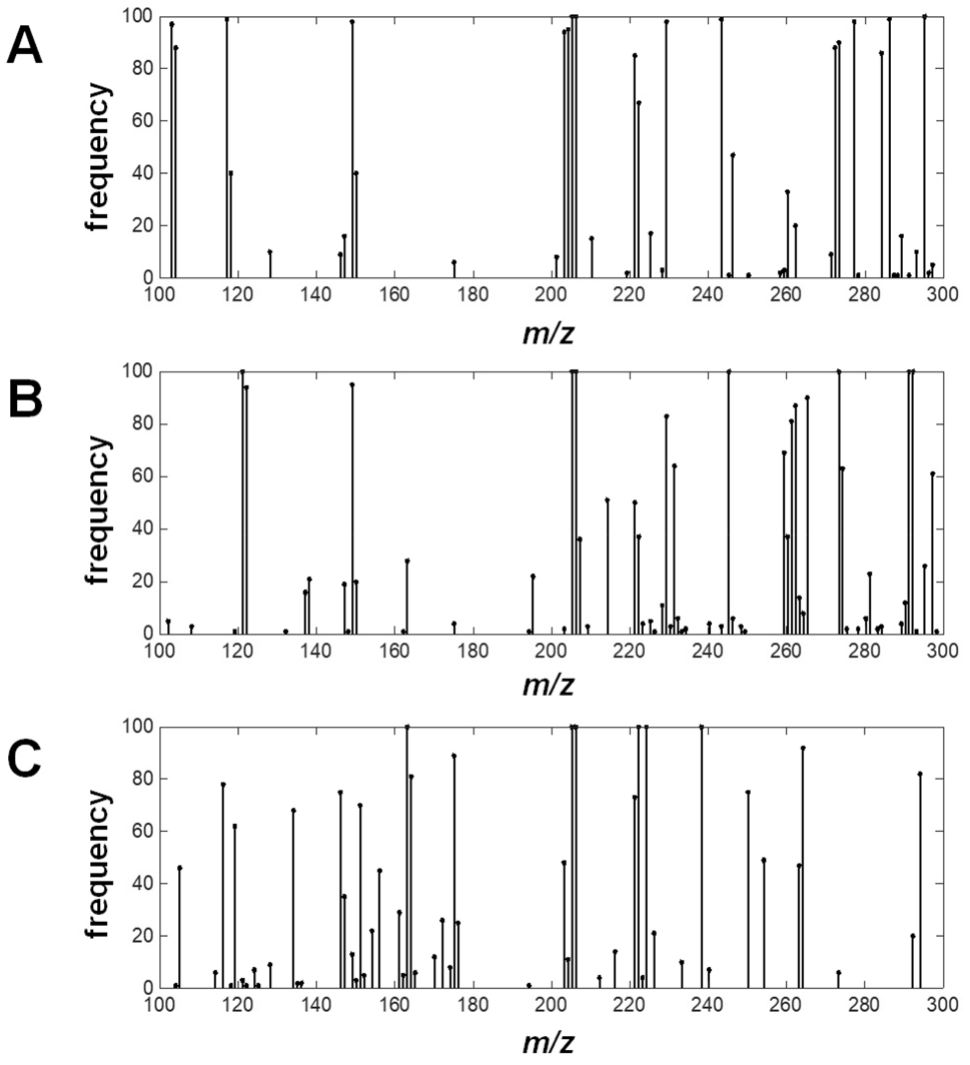
Bar charts showing the number of times each variable (*m/z* data points) was found in 100 iterations in the studies about (**A**) the type of peppers, (**B**) the origins of black peppers, and (**C**) the origins of white peppers.

Figure 6 illustrates the number of times (out of 100 iterations) that each *m/z* value was selected in the subset of the top 10% *m/z* data points with the highest magnitude of Fisher weight value. Any *m/z* values that are present for 100 times were identified as impactful markers. The study on the types of peppers provides a set of 3 *m/z* data points (*m/z* = 205.2, 206.2 and 295.2) that appeared in all 100 iterations (Fig. 6A), while the study about the origins of white peppers gave only 6 *m/z* data points (*m/z* = 163.1, 205.2, 206.2, 222.2, 224.2, and 238.3) that appeared 100 times (Fig. 6C). In the case of the study about the origins of black peppers, there were 7 *m/z* values (121.1, 205.2, 206.2, 245.2, 273.3, 291.2, 292.1, 292.2) that appeared in all 100 iterations (Fig. 6B), which indicated a more challenging case due to higher numbers of variables. It can be seen that in all cases, *m*/*z* 205.2 appeared as a significant marker, which is attributed to sesquiterpenes, a major class of compounds found in peppercorn seeds. It should be noted, however, that focusing on individual data points may be misleading due to the possibility for strategic adulteration/modification. This is actually reflected in the results, where different studies required different numbers of variables for effective cluster separations. Therefore, the non-targeted approach^56^, as practiced in this study, is deemed to be more flexible and versatile for broader applications. This is because it is not subject to strategic adulteration from a selected small set of known markers.

## Conclusion

In this study, a simple and rapid MS-based method to reveal chemical profiles was demonstrated that is capable of distinguishing geographical origins. In particular, corona discharge was used to ionize volatile compounds from peppercorns for subsequent MS analysis. With its relatively simple setup, the method allowed for rapid data collection, which can then be further processed by chemometrics. Such an approach has potential to be used as part of a portable setup for remote and onsite analysis. After pre-processing, linear discriminant analysis indicated that this experimental setup had high discrimination efficiency (> 98% accuracies) in all studies involving the type of peppers, the origins of black peppers, and the origins of white peppers. This positive outcome suggests that this method can be applied to many more cases where whole agricultural products are directly analyzed for rapid discrimination of origins and without any sample pretreatment or workup.

## Data availability

A set of supplementary information is available online including an overview scheme of data preparation process, Preliminary and comparative PS-MS data, MS/MS data, Representative MS spectra for all samples, Prediction strength values for all studies, Classification rates, and Raw numerical data in a spreadsheet.

## Supplementary Information

Supplementary Information 1.

Supplementary Information 2.

## References

[1] Maher S, Jjunju FPM, Taylor S (2015). Colloquium: 100 years of mass spectrometry: Perspectives and future trends. Rev. Mod. Phys..

[2] Smith BL (2019). Rapid scotch whisky analysis and authentication using desorption atmospheric pressure chemical ionisation mass spectrometry. Sci. Rep..

[3] Jeng J-Y (2020). Obtaining molecular imagings of pesticide residues on strawberry surfaces with probe sampling followed by ambient ionization mass spectrometric analysis. J. Mass Spectrom..

[4] Chen S (2017). Rapid analysis of bisphenol a and its analogues in food packaging products by paper spray ionization mass spectrometry. J. Agric. Food Chem..

[5] Feider CL, Krieger A, DeHoog RJ, Eberlin LS (2019). Ambient Ionization Mass Spectrometry: Recent Developments and Applications. Anal. Chem..

[6] Damon DE (2016). 2D wax-printed paper substrates with extended solvent supply capabilities allow enhanced ion signal in paper spray ionization. Analyst.

[7] Suraritdechachai S (2019). Rapid detection of the antibiotic sulfamethazine in pig body fluids by paper spray mass spectrometry. J. Agric. Food Chem..

[8] Damon DE (2019). Determining Surface Energy of Porous Substrates by Spray Ionization. Langmuir.

[9] Sarih NM (2020). Accelerated nucleophilic substitution reactions of dansyl chloride with aniline under ambient conditions via dual-tip reactive paper spray. Sci. Rep..

[10] Liu J (2010). Development, characterization, and application of paper spray ionization. Anal. Chem..

[11] Gross JH (2014). Direct analysis in real time—A critical review on DART-MS. Anal. Bioanal. Chem..

[12] Marić M, Marano J, Cody RB, Bridge C (2018). DART-MS: A new analytical technique for forensic paint analysis. Anal. Chem..

[13] Forbes TP, Sisco E, Staymates M, Gillen G (2017). DART-MS analysis of inorganic explosives using high temperature thermal desorption. Anal. Methods.

[14] Ifa DR, Wu C, Ouyang Z, Cooks RG (2010). Desorption electrospray ionization and other ambient ionization methods: Current progress and preview. Analyst.

[15] Garza KY (2018). Desorption electrospray ionization mass spectrometry imaging of proteins directly from biological tissue sections. Anal. Chem..

[16] Smith MJP, Cameron NR, Mosely JA (2012). Evaluating Atmospheric pressure Solids Analysis Probe (ASAP) mass spectrometry for the analysis of low molecular weight synthetic polymers. Analyst.

[17] Tose LV, Murgu M, Vaz BG, Romão W (2017). Application of atmospheric solids analysis probe mass spectrometry (ASAP-MS) in petroleomics: Analysis of condensed aromatics standards, crude oil, and paraffinic fraction. J. Am. Soc. Mass Spectrom..

[18] Jjunju FPM (2015). Analysis of polycyclic aromatic hydrocarbons using desorption atmospheric pressure chemical ionization coupled to a portable mass spectrometer. J. Am. Soc. Mass Spectrom..

[19] Jjunju FPM (2015). Hand-held portable desorption atmospheric pressure chemical ionization ion source for in situ analysis of nitroaromatic explosives. Anal. Chem..

[20] Jafari MT, Khayamian T, Shaer V, Zarei N (2007). Determination of veterinary drug residues in chicken meat using corona discharge ion mobility spectrometry. Anal. Chim. Acta.

[21] Smith BL (2020). Flexible drift tube for high resolution ion mobility spectrometry (Flex-DT-IMS). Anal. Chem..

[22] Song L, You Y, Perdomo NR, Evans-Nguyen T (2020). Inexpensive ultrasonic nebulization coupled with direct current corona discharge ionization mass spectrometry for liquid samples and its fundamental investigations. Anal. Chem..

[23] Mullen M, Giordano BC (2020). Combined secondary electrospray and corona discharge ionization (SECDI) for improved detection of explosive vapors using drift tube ion mobility spectrometry. Talanta.

[24] Sekimoto K (2016). Improvement in ionization efficiency of direct analysis in real time-mass spectrometry (DART-MS) by corona discharge. Analyst.

[25] Chingin K (2016). Rapid detection of Mycobacterium tuberculosis cultures by direct ambient corona discharge ionization mass spectrometry of volatile metabolites. RSC Adv..

[26] Hu L (2016). Early release of 1-pyrroline by *Pseudomonas aeruginosa* cultures discovered using ambient corona discharge ionization mass spectrometry. RSC Adv..

[27] Zhang X (2018). Deciphering the chemical origin of the semen-like floral scents in three angiosperm plants. Phytochemistry.

[28] Hang Y, Chingin K, Liang J, Wang X, Hu L (2017). Fast detection of volatile organic compounds from Staphylococcal blood cultures by CDI-MS. RSC Adv..

[29] FAO. *FAOSTAT*, http://www.fao.org/faostat/en/#data/TP (8 March 2021).

[30] Wang P, Yu Z (2015). Species authentication and geographical origin discrimination of herbal medicines by near infrared spectroscopy: A review. J. Pharm. Anal..

[31] Lawless LJR, Hottenstein A, Ellingsworth J (2012). The Mccormick spice wheel: A systematic and visual approach to sensory lexicon development. J. Sens. Stud..

[32] Witkowska AM, Hickey DK, Alonso-Gomez M, Wilkinson MG (2011). The microbiological quality of commercial herb and spice preparations used in the formulation of a chicken supreme ready meal and microbial survival following a simulated industrial heating process. Food Control.

[33] Tripathy V, Basak BB, Varghese TS, Saha A (2015). Residues and contaminants in medicinal herbs—A review. Phytochem. Lett..

[34] Wilde AS, Haughey SA, Galvin-King P, Elliott CT (2019). The feasibility of applying NIR and FT-IR fingerprinting to detect adulteration in black pepper. Food Control.

[35] Lafeuille J-L, Frégière-Salomon A, Michelet A, Henry KL (2020). A Rapid non-targeted method for detecting the adulteration of black pepper with a broad range of endogenous and exogenous material at economically motivating levels using micro-ATR-FT-MIR imaging. J. Agric. Food Chem..

[36] Lima ABS (2020). Fast quantitative detection of black pepper and cumin adulterations by near-infrared spectroscopy and multivariate modeling. Food Control.

[37] Liang J (2021). Chemical analysis and classification of black pepper (*Piper nigrum* L.) based on their country of origin using mass spectrometric methods and chemometrics. Food Res. Int..

[38] Josling T (2006). The war on terroir: Geographical indications as a transatlantic trade conflict. J. Agric. Econ..

[39] WTO. *Intellectual property (TRIPS) - Geographical indications - Background and the current situation*, https://www.wto.org/english/tratop_e/trips_e/gi_background_e.htm (13 March 2021).

[40] EU. *Geographical Indications—a European treasure worth €75 bn*, https://ec.europa.eu/commission/presscorner/detail/en/IP_20_683 (8 March 2021).

[41] Luykx DMAM, van Ruth SM (2008). An overview of analytical methods for determining the geographical origin of food products. Food Chem..

[42] Dias C, Mendes L (2018). Protected designation of origin (PDO), protected geographical indication (PGI) and traditional speciality guaranteed (TSG): A bibiliometric analysis. Food Res. Int..

[43] Consonni, R. & Cagliani, L. R. in *Advances in Food and Nutrition Research* Vol. 59 (ed Steve L. Taylor) 87–165 (Academic Press, 2010). 10.1016/S1043-4526(10)59004-1 20610175

[44] Brereton RG (2003). Chemometrics: Data Analysis for the Laboratory and Chemical Plant.

[45] Brereton RG (2007). Applied Chemometrics for Scientists.

[46] Brereton RG (2009). Chemometrics for Pattern Recognition.

[47] Wongravee K (2009). Variable selection using iterative reformulation of training set models for discrimination of samples: Application to gas chromatography/mass spectrometry of mouse urinary metabolites. Anal. Chem..

[48] Ravindran, P. N. & Kallupurackal, J. A. in *Handbook of Herbs and Spices* (ed K. V. Peter) 86–115 (Woodhead Publishing, 2012).

[49] Silvis ICJ, Luning PA, Klose N, Jansen M, van Ruth SM (2019). Similarities and differences of the volatile profiles of six spices explored by Proton Transfer Reaction Mass Spectrometry. Food Chem..

[50] Singh G, Marimuthu P, Catalan C, de Lampasona M (2004). Chemical, antioxidant and antifungal activities of volatile oil of black pepper and its acetone extract. J. Sci. Food Agric..

[51] Jirovetz L, Buchbauer G, Ngassoum MB, Geissler M (2002). Aroma compound analysis of *Piper nigrum* and *Piper guineense* essential oils from Cameroon using solid-phase microextraction–gas chromatography, solid-phase microextraction–gas chromatography–mass spectrometry and olfactometry. J. Chromatogr. A.

[52] Kapoor IPS (2009). Chemistry and in vitro antioxidant activity of volatile oil and oleoresins of black pepper (*Piper nigrum*). J. Agric. Food Chem..

[53] Li Y-X (2020). Analysis of chemical components and biological activities of essential oils from black and white pepper (*Piper nigrum* L.) in five provinces of southern China. LWT.

[54] PubChem. *delta-Elemene|C15H24-PubChem*, https://pubchem.ncbi.nlm.nih.gov/compound/delta-Elemene?fbclid=IwAR2qQEQ8aWK2-rVj8wUBYMZFQTjI-J2uP8pm6vrZHIrRATFhd0da8ECnUlU (7 July 2021).

[55] Wongravee K (2009). Monte-Carlo methods for determining optimal number of significant variables. Application to mouse urinary profiles. Metabolomics.

[56] Esslinger S, Riedl J, Fauhl-Hassek C (2014). Potential and limitations of non-targeted fingerprinting for authentication of food in official control. Food Res. Int..

