# Supplementary material for: Rapid geographical indication of peppercorn seeds using corona discharge mass spectrometry
Source: Sci Rep. 2021 Aug 9;11:16089. doi: 10.1038/s41598-021-95462-0 (PMC8352875; doi:10.1038/s41598-021-95462-0)
Supplement: Supplementary file 2 — Supplementary Information 2. [file 41598_2021_95462_MOESM2_ESM.pdf]

SUPPLEMENTARY INFORMATION

Rapid Geographical Indication of Peppercorn Seeds using Corona Discharge Mass Spectrometry

Preeyarad Charoensumran,[a,b] Monrawat Rauytanapanit,[a,b] Nontawat Sricharoen,[a,c] Bary L. Smith,[e] Kanet Wongravee,[a,d*] Simon Maher,[e] and Thanit Praneenararat[a,b,*]

[a]Department of Chemistry, Faculty of Science, Chulalongkorn University, Phayathai Rd., Pathumwan, Bangkok, 10330, Thailand.

[b]The Chemical Approaches for Food Applications Research Group, Faculty of Science, Chulalongkorn University, Phayathai Rd., Pathumwan, Bangkok, 10330, Thailand.

[c]Center of Excellence in Bioactive Resources for Innovative Clinical Applications, Chulalongkorn University, Bangkok 10330, Thailand.

[d]Sensor Research Unit, Department of Chemistry, Faculty of Science, Chulalongkorn University, Phayathai Rd., Pathumwan, Bangkok, Thailand, 10330.

[e]Department of Electrical Engineering & Electronics, University of Liverpool, Brownlow Hill, Liverpool, L69 3GJ, United Kingdom.

***Corresponding Author**

Email: Thanit.P@chula.ac.th; Tel: +66-2-218-7638

Email: Kanet.W@chula.ac.th; Tel: +66-2-218-7589

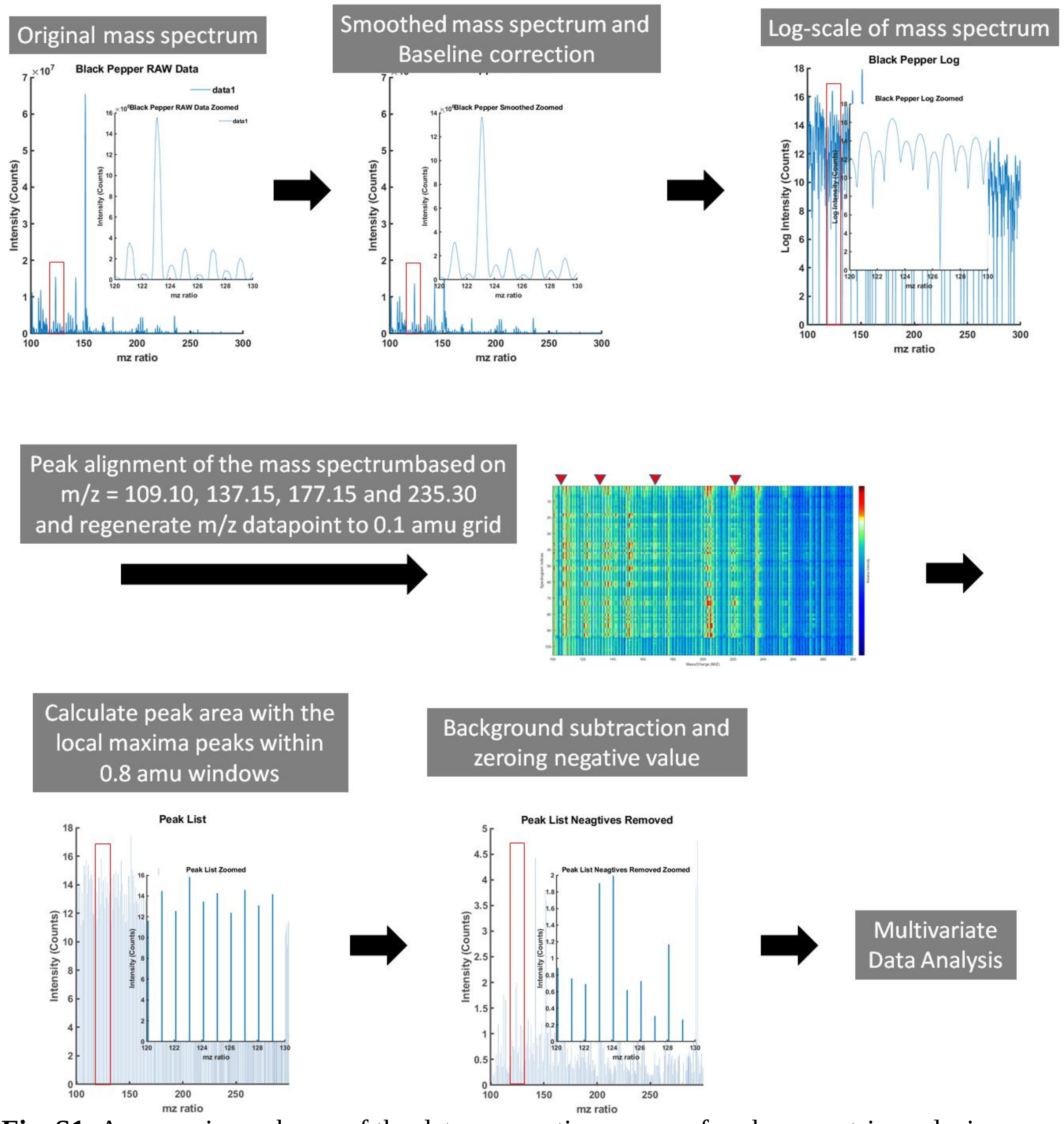

Fig. S1. An overview scheme of the data preparation process for chemometric analysis.

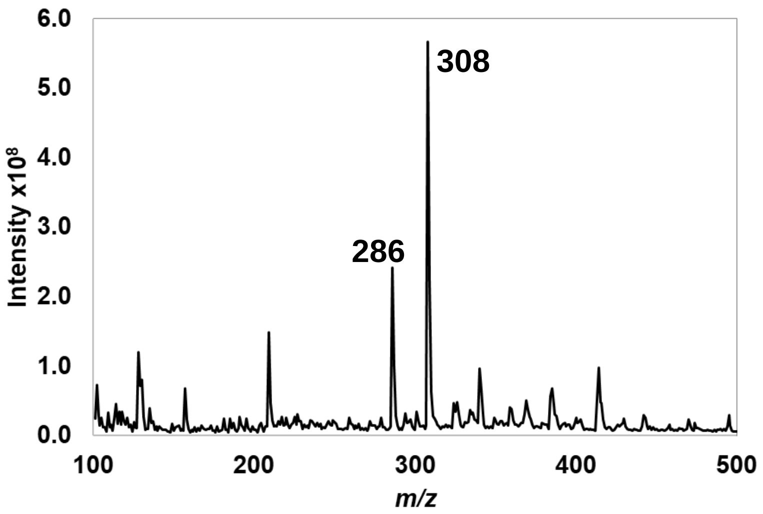

Fig. S2. Preliminary PS-MS data of an aqueous extract of black pepper. $[M+H]^+$ and $[M+Na]^+$ indicative of piperine at *m*/*z* 286 and *m*/*z* 308, respectively, are clearly visible.

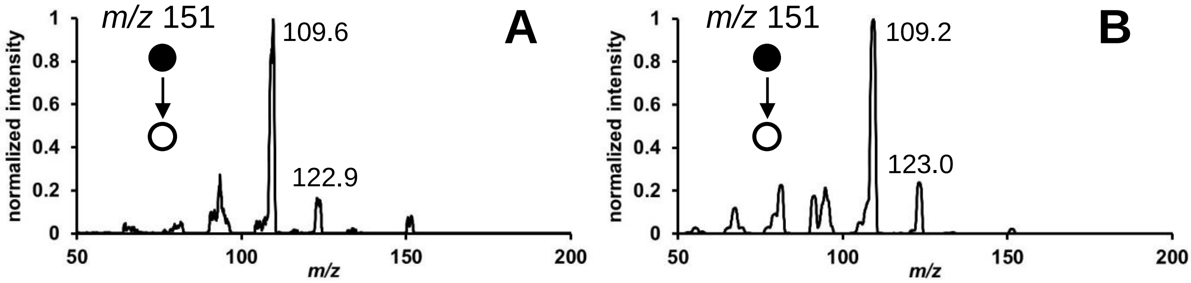

Fig. S3. Comparison of A) CD-MS/MS spectrum from black pepper, and B) direct-infusion MS/MS spectrum of (*R*)-carvone. The parent ion in both cases was *m/z* 151 (collision energy = 18 V).

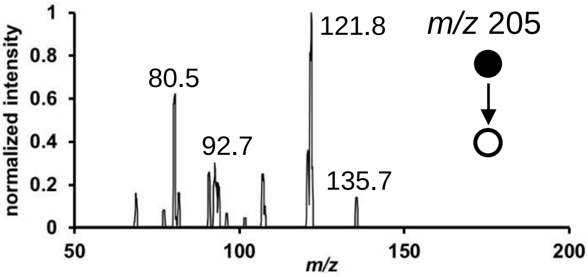

Fig. S4. CD-MS/MS spectrum from black pepper at a parent ion of *m/z* 205.

Fig. S4 shows the daughter ion scan following collision induced dissociation (CID) of *m/z* 205 (collision energy = 20 V) direct from a black peppercorn seed. In the absence of δ-elemene MS/MS data for direct comparison, an EI spectrum was used (ref. 54 in the main article). Good similarity was obtained, in particular commonality was observed at peaks: *m/z* 93, 121 and 136. Therefore, based on this data and its well-known presence in black pepper (ref. 50 in the main article), we tentatively suggest that this peak is from δ-elemene (or its isomers).

A: monoterpene, B: carvone, C: sesquiterpene

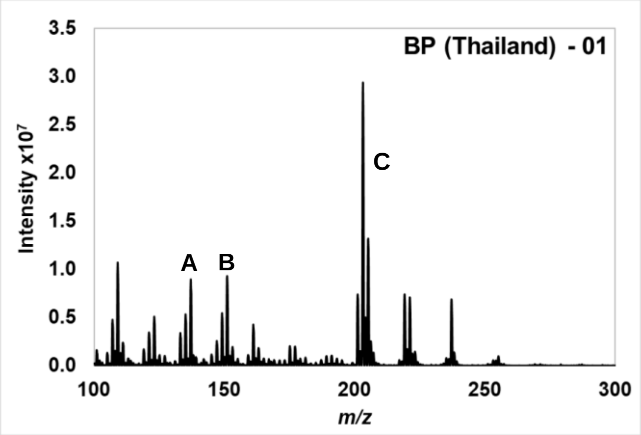

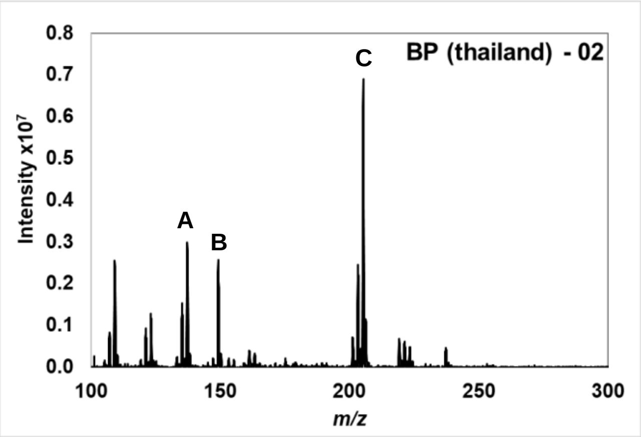

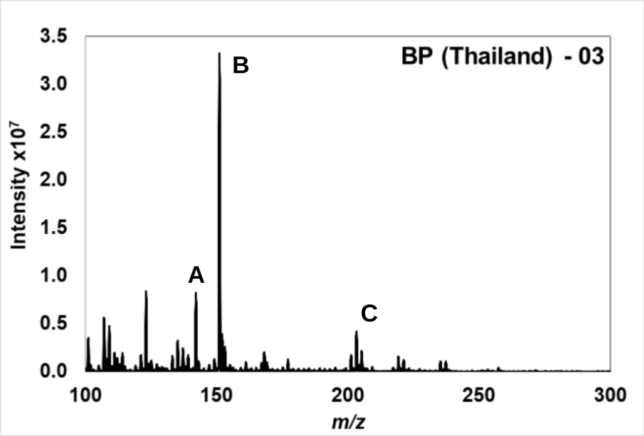

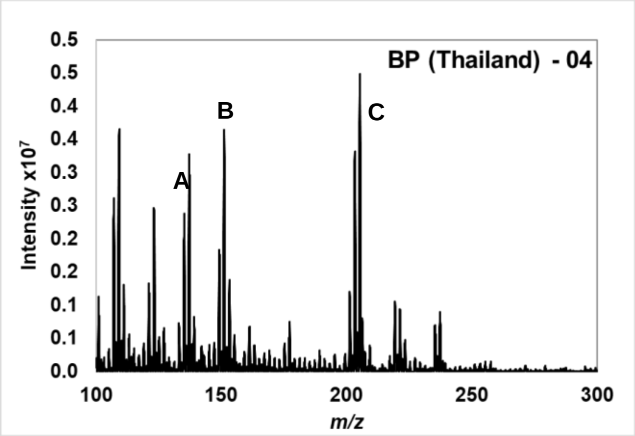

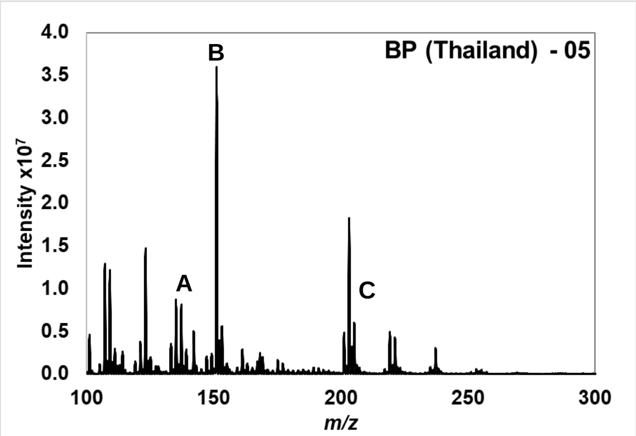

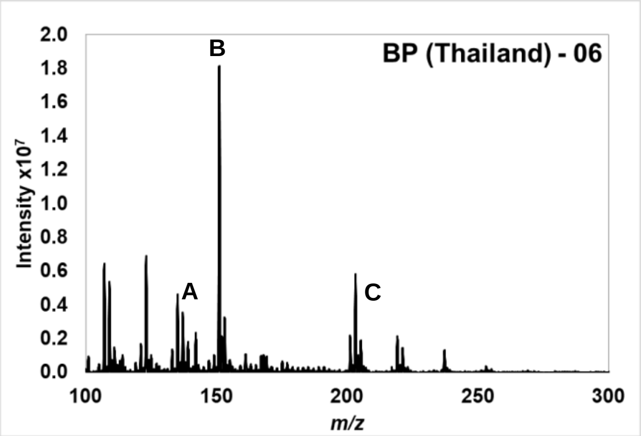

A: monoterpene, B: carvone, C: sesquiterpene

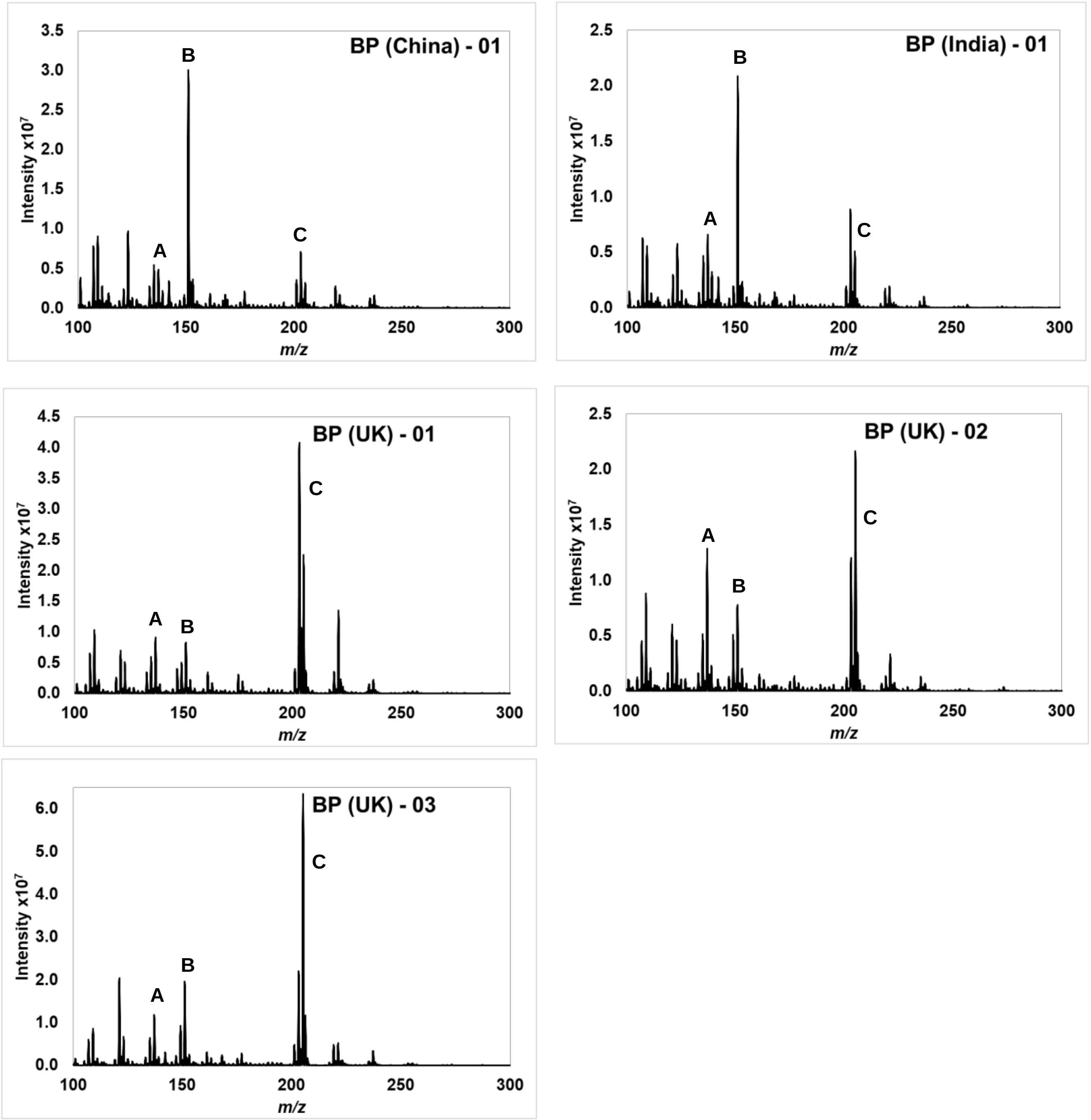

Fig. S5. Representative MS spectra of all black pepper samples.

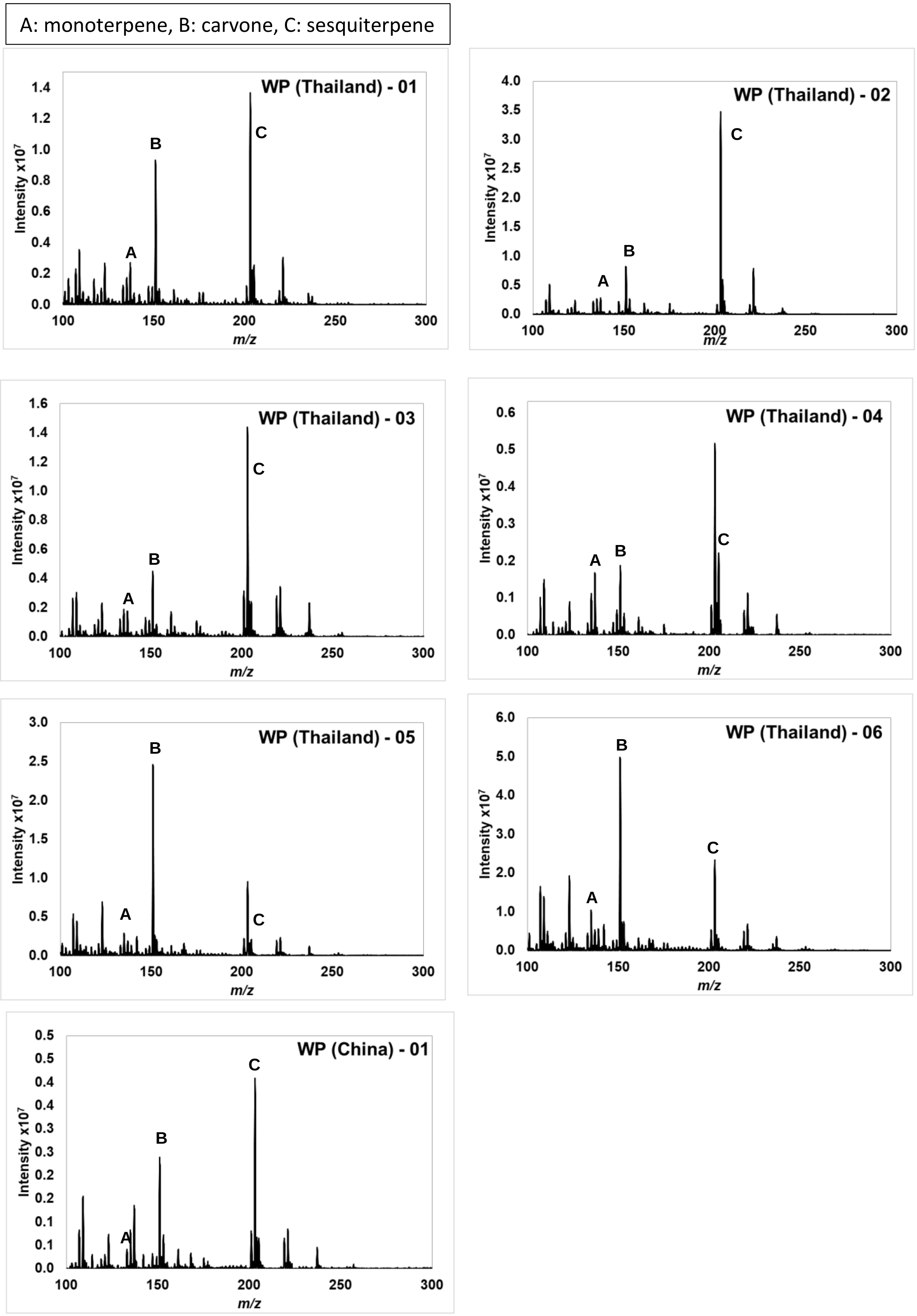

Fig. S6. Representative MS spectra of all white pepper samples.

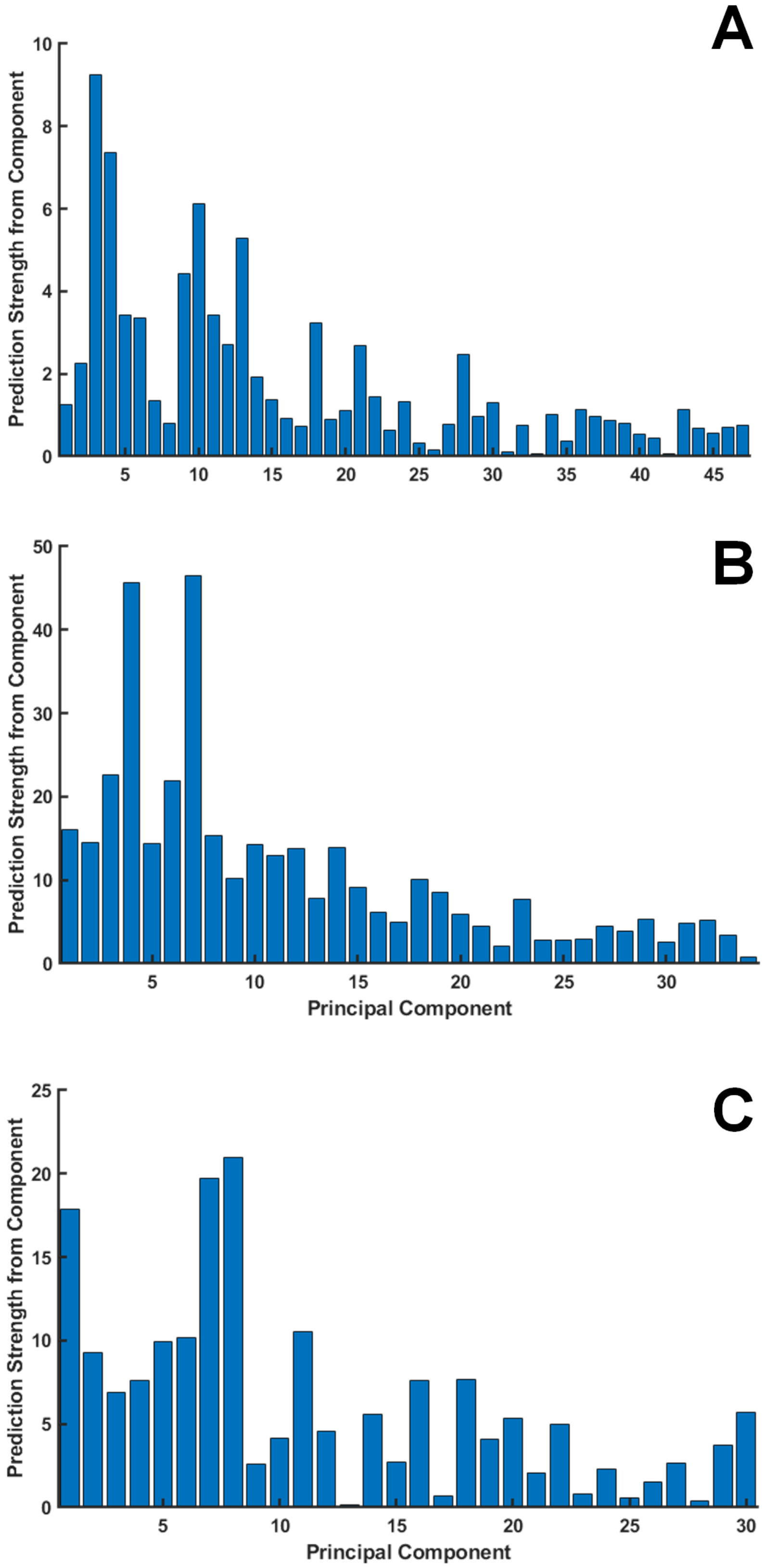

Fig. S7. Prediction Strength in A) the study of the types of peppers; B) the study of geographical origins of black peppers; and C) the study of geographical origins of white peppers

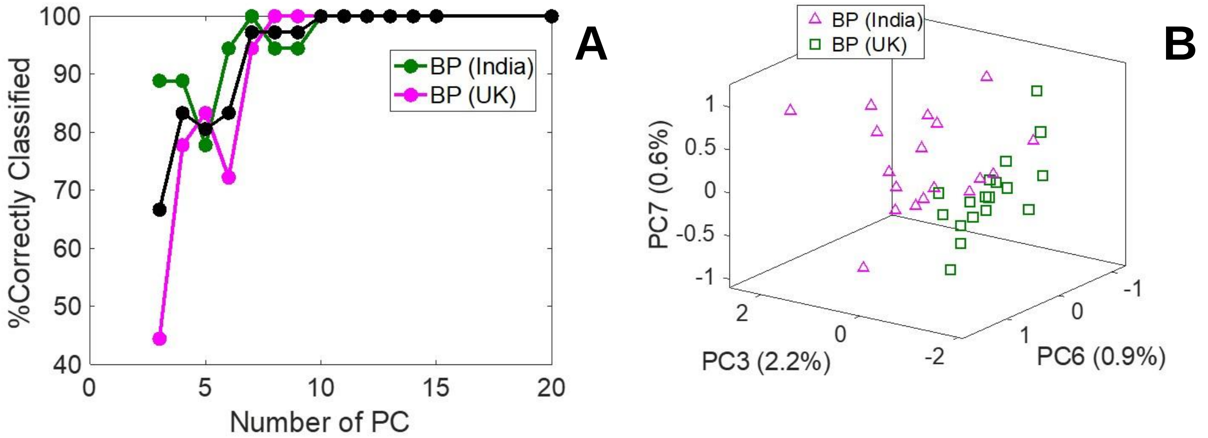

Fig. S8. A) The percentage of correctly classified samples (India vs UK) with the number of PCs used to build a model (PC1-20) in an exemplary PS-MS experiment of the aqueous extracts of black peppers. B) The respective PC score plots using the best discriminate PCs including PC3, PC6, and PC7.

Table S1. Comparison of the classification rates from first three PCs and the optimized numbers of PCs in the studies of A) types of peppers (18 PCs), B) origins of black peppers (14 PCs), and C) origins of white peppers (11 PCs).

A

First 3 PCs		True class	
		Black pepper	White pepper
Predict class	Black pepper	76 (80.85%)	18
	White pepper	18	44 (70.97%)
	Sum	94	62

18 PCs		True class	
		Black pepper	White pepper
Predict class	Black pepper	93 (98.94%)	1
	White pepper	1	61 (98.39%)
	Sum	94	62

B

First 3 PCs		True class			
		BP (Thailand)	BP (China)	BP (India)	BP (UK)
Predict class	BP (Thailand)	21 (42%)	3	0	4
	BP (China)	18	3 (33.33%)	4	0
	BP (India)	9	3	3 (33.33%)	7
	BP (UK)	2	0	2	15 (57.69%)
	Sum	50	9	9	26

14 PCs		True class			
		BP (Thailand)	BP (China)	BP (India)	BP (UK)
Predict class	BP (Thailand)	50 (100%)	0	0	1
	BP (China)	0	9 (100%)	0	0
	BP (India)	0	0	9 (100%)	0
	BP (UK)	0	0	0	25 (96.15%)
	Sum	50	9	9	26

C

First 3 PCs		True class	
		WP (Thailand)	WP (China)
Predict class	WP (Thailand)	37 (69.81%)	1
	WP (China)	16	8 (88.89%)
	Sum	53	9

11 PCs		True class	
		WP (Thailand)	WP (China)
Predict class	WP (Thailand)	53 (100%)	0
	WP (China)	0	9 (100%)
	Sum	53	9
